# Specialist follow-up contraceptive support after abortion—Impact on effective contraceptive use at six months and subsequent abortions: A randomised controlled trial

**DOI:** 10.1371/journal.pone.0217902

**Published:** 2019-06-11

**Authors:** Usha Kumar, Louise Pollard, Lucy Campbell, Selin Yurdakul, Clara Cantalapiedra Calvete, Bola Coker, Tao Chen, Abdel Douiri

**Affiliations:** 1 Department of Sexual Health and HIV, King’s College Hospital NHS Foundation Trust, London, United Kingdom; 2 Academic Department of Sexual Health and HIV, King’s College London, Weston Education Centre, London, United Kingdom; 3 Department of Primary Care and Public Health Sciences, King's College London, Addison House, Guy’s Campus, London, United Kingdom; 4 King’s College London, London, United Kingdom; 5 NIHR Biomedical Research Centre, Guy’s and St.Thomas’ NHS Foundation Trust and King’s College London, United Kingdom; University of Washington, UNITED STATES

## Abstract

**Objectives:**

To assess the impact of specialist contraceptive support after abortion on effective contraceptive use at six months and subsequent abortions within two years.

**Methods:**

Multicentre randomised controlled trial among women undergoing induced abortion in three London boroughs. Allocation was through electronically concealed stratified randomisation by centre, blinding clinicians and participants to arm allocation until interventions. Control group received standard care, comprising advice to follow up with their general practitioner or contraceptive clinic as needed. Intervention group additionally received specialist contraceptive support via telephone or face-to-face consultation at 2–4 weeks and 3 months post-abortion. Primary outcome was rate of effective contraceptive use at six months post-abortion. Secondary outcomes were subsequent abortions within two years.

**Results:**

569 women were recruited between October 2011 and February 2013, randomised to intervention (282) and control (287) groups; 290 (142 intervention, 148 control) were available for primary outcome analysis. Intention-to-treat analysis showed no significant difference between the two groups in effective contraceptive use after abortion (62%, vs 54%, p = 0·172), long-acting contraceptive use (42% versus 32%, p = 0·084), and subsequent abortion (similar rates, at 1 year: 10%, p = 0·895, between 1–2 years: 6%, p = 0·944). Per-protocol analysis showed those who received the complete intervention package were significantly more likely to use effective contraception (67% versus 54%, p = 0·048), in particular long-acting contraception (49% versus 32%, p = 0·010) and showed a non-significant reduction in subsequent abortions within 2 years (at 1 year: 5% versus 10%, p = 0·098; and between 1–2 years: 3% versus 6%, p = 0·164, respectively).

**Conclusions:**

Structured specialist support post-abortion did not result in significant use of effective contraception at six months or reduction in subsequent abortions within two years. Participants engaging with the intervention showed positive effect on effective contraception at six months post-abortion. The potential benefit of such intervention may become evident through further studies with increased patient participation.

## Introduction

Abortion rates in England and Wales have declined in the last decade, however subsequent abortions are on the rise; in 2016, 38% of women undergoing abortions had one or more previous abortions as compared to 32% in 2006 [1]. In London alone 42% of the abortions in 2016 were subsequent abortions [1], despite the widespread availability of free contraception. National population-level data on abortions within two years after an initial abortion are not available, however, hospital-level data from studies in Scotland have reported rates ranging from 12·3% [2] to 14·6% [3]. Pregnancies ending in subsequent abortions within a short timeframe are likely to be unintended, reflecting unmet contraceptive needs and highlighting the gap in provision of effective post-abortion care.

Previous studies showed that contraceptive risk-taking is evenly high before and after abortion, and peri-abortion contraceptive counselling may be ineffective or inadequate [4,5]. Contraceptive needs of women undergoing abortion are often complex due to various psycho-social factors [6]. Effective contraceptive counselling in abortion clinics is challenging, particularly within the time available to healthcare professionals during the abortion consultation [5,7–9]. In addition, women need to process a large amount of information [8,10] and make informed decisions during a potentially emotionally stressful period.

Randomised controlled trials (RCT) of peri-abortion contraception counselling interventions show mixed results [3,8,10–13]. Recent systematic reviews on this subject reported no evidence of enhanced counselling on uptake of long-acting reversible contraception (LARC) or subsequent pregnancy [14,15]. However, significant clinical heterogeneity was observed between all the studies [15].

Care pathways to improve post-abortion contraception have been recommended [16,17]. In practice, these rely predominantly on self-referrals. Attendance rates at routine post-abortion follow-up appointments as low as 50% have been reported [3,18]. Women may not seek professional advice if there are no problems related to the abortion itself. However, focused patient-centred peri-abortion contraceptive counselling with structured follow-up could potentially enhance contraceptive use, as suggested by several authors in the literature [19–21].

We aimed to assess the effect of structured specialist contraceptive support provided during the three-month period following abortion and hypothesised that this would facilitate initiation and continuation of effective contraception and reduce subsequent abortions within two years.

## Methods

### Design

A multicentre randomised parallel-group controlled trial was conducted at the three abortion clinics located in the South London boroughs of Lambeth, Southwark and Lewisham (LSL) that provide services for the National Health Service (NHS). Participating centres included British Pregnancy Advisory Service (BPAS), Marie Stopes International (MSI), and King’s College Hospital. A stratified design, which divided the sample among 6 strata (3 centres, 2 arms in each centre) was used for the study. Patients were recruited over a 16 month period between October 2011 and February 2013. Follow-up period commenced in October 2011 and finished in February 2015 which included the primary and secondary outcome follow-up period.

Ethical approval for the study was obtained from the National Research Ethics committee on 22^nd^ March 2011 and the respective ethics committees of BPAS and MSI. The study was entered on to the National Institute for Health Research Clinical Research Network (NIHR CRN) portfolio in July 2011. The study was registered with the ISRCTN Registry, number ISRCTN 95625309 (http://www.isrctn.com/ISRCTN95625309). There was an unintended gap of 28 days from date of first patient recruitment to ISRCTN registration date. The research team were not aware at that time, of the recommendation for prospective registration of the trial before first patient recruitment. This has not resulted in any reporting bias for the results of the study.

### Participants

Women of all ages, either resident or registered with a general practitioner (GP) in the LSL area seeking an abortion funded by the NHS were eligible for participation in the study. Patients were excluded if they could not speak English, intended to leave United Kingdom within six months following the abortion, lacked capacity to consent for themselves or decided to continue with the pregnancy. Participants under the age of 16 years were assessed for their capacity to understand fully the specific circumstances and the details of the research being proposed and their ability to use and weigh this information in reaching a decision (‘Gillick Competence’) for participation in the study. Participants who were deemed to lack capacity to consent were excluded from the study as per the trial protocol. We did not obtain informed consent from the next of kin, caretakers, or guardians on behalf of the minors/children enrolled in the study. This approach was approved by the Ethics committee.

Women seeking abortion have to use a central telephone booking service in Lambeth, Southwark and Lewisham. They were first informed about the study when booking their appointment for the abortion clinic and written information was sent when they expressed an interest. Patients were screened for eligibility when they attended their booked appointment. Eligible participants were identified, and further information was provided about the research on the day of their appointment. After their abortion consultation, written informed consent was obtained from all participants. Baseline demographic, obstetric and contraception data (Topic Guide section A in S1 Topic Guide) were obtained following enrolment. There was no financial motivation for participation or continuation in our study as contraceptives were available free of cost.

### Randomisation and masking

Participants were randomised to either standard practice or structured specialist contraceptive support for three months after abortion. Randomisation was stratified by centre to ensure any between clinic variance was taken into account in the randomisation procedure. Participants were randomised 1:1 to intervention and control groups within each clinic. We used block randomisation with varying block sizes from four to twelve. A computer-generated randomisation list was prepared which was concealed and embedded in a dedicated online electronic database. The allocation sequence was concealed from the person enrolling and assessing participants to the study. Participants were randomised chronologically when enrolment data was uploaded to the database by a member of the research team. Participants were blinded to arm allocation until interventions. Clinicians providing the intervention and assessing outcomes were not blinded after assignment to the arms. To provide continuity of care, both interventions and data collection were carried out by the same specialist research nurse, trained in contraception and sexual health.

### Intervention and control

In the area where we conducted our study, contraceptive counselling and provision of free contraception including LARC methods at the time of abortion care formed part of NHS service contracts for abortion providers. Post-abortion follow-up is not routinely organised and patients are advised to see their general practitioner (GP) or contraceptive service provider for follow-up as required. This constituted standard care for this study.

The intervention arm additionally received two follow-up contacts from the research nurse via telephone for contraceptive support at two-to-four weeks and three months post-abortion. Participants received individualised contraceptive counselling which explored psycho-social, personal, contraception method-related, partner-related and medical factors that could influence contraceptive decision-making, compliance and continuation. Contraceptive initiation and continuation was further facilitated through direct referral to specialist contraceptive services as needed. Patients requesting a face-to-face appointment had the opportunity to see a consultant in sexual & reproductive healthcare. A questionnaire (Topic Guide section B, C and D in S1 Topic Guide) was used during the follow-up to structure the interviews, and information collected was recorded in an online case report form (CRF). When patients were undecided on a contraception plan after the initial follow-up, further follow-up consultations were arranged as necessary to allow sufficient time for decision-making.

Intervention and control participants were contacted at six months post-abortion by the same research nurse for data collection only. Information about contraceptive use, compliance, satisfaction with their chosen contraceptive method, the number of self-initiated contacts with health professionals seeking contraceptive advice and pregnancy intentions was recorded using a questionnaire (Topic Guide section E in S1 Topic Guide).

At the six-month interview, for the primary outcome assessment, the research nurse used a structured compliance assessment framework to assess whether participants reporting use of oral contraceptive pills /patches/vaginal ring/injectable contraception were using their contraceptive method regularly and consistently. This framework was based on the standard advice provided to patients in the UK Family Planning Association Contraception leaflets. For methods such as oral contraceptive pills/patches/vaginal ring, participants were asked to describe how they were using the method, and if they were following the correct recommended schedule. Similarly, for injectable contraception, the nurse checked if they were adhering to the recommended injection intervals. If there were any deviations from the recommended schedule, or if they were taking any medications that could affect the efficacy of the methods, the nurse checked whether they were using any additional precautions. Patients chose the method of contact at recruitment. The majority (96%) chose to be contacted by mobile phone. A maximum of four attempts were made at each contact point to reduce loss-to-follow-up. The research nurse also offered flexibility of timing for contact, which included evening and weekend consultations.

Data on subsequent abortions for the study patients at one and two years following abortion was obtained through data linkage with the national abortion dataset for England and Wales routinely collected by the Department of Health (DH) through statutory notification. With patient consent, date of birth and postcode were used to collect information about subsequent abortions.

### Primary and secondary outcomes

The primary outcome of the study was the rate of effective contraceptive use at six months post-abortion; effective contraceptive use being defined as regular and consistent use of oral contraceptive pills/patches/vaginal ring/injectable contraception, or the use of intrauterine contraception or the contraceptive implant, or sterilisation. Barrier contraception, abstinence, no method, withdrawal method, natural family planning and suboptimal use of user-dependent hormonal methods were defined as “non-effective” contraception for the purpose of this study. The secondary outcomes were incidence of subsequent abortion within one and two years following the index abortion.

No adverse events from the intervention package were reported. Where issues such as alcohol/drug abuse, domestic violence, sexual exploitation, child protection, psychiatric disorders, and significant psychological distress were identified, referrals to appropriate agencies were made.

### Sample size

We calculated that sample sizes, summed across all strata, of 223 in intervention group and 223 in control group would achieve 95% power and 5% significance to detect a difference of 15% in the percentage of women using effective contraception in each group at six months’ follow-up. We anticipated a 65% participation rate and 60% follow-up rate based on a previous study [3], and calculated that we needed to screen 1144 patients across the three centres and recruit 743 patients, to expect 446 patients to be available for the primary outcome analysis at 6 months. The preplanned sample size was not achieved as two of the centres were unable to continue participation until target numbers could be reached. 1006 patients were screened, 569 were recruited (57% participation rate) and 290 (142 in the intervention arm and 148 in the control arm) were available at 6 months for primary outcome analysis which reduced the power of the study to 81%.

### Statistical analysis

Summary statistics are presented as percentages for categorical variables and as means with standard deviations for continuous variables. Continuous variables were compared by unpaired t-test and the Pearson chi-square test was used to compare proportions. Data from all randomised participants who were available at six-month follow-up, were used for primary outcome analysis by intention-to-treat. As randomisation was performed by centre, additional analysis with adjustment by centre was tested. All randomised participants were included in the one- and two-year subsequent abortion analysis, by intention-to-treat. Per-protocol analyses were conducted on women who received the complete intervention. Logistic regression was used to assess the change in contraceptive use prior to abortion to six months post-abortion. A p-value of less than 0·05 was considered statistically significant. All analyses were made using STATA version 14 software (StataCorp LP, College Station, TX). A data monitoring committee oversaw the study.

## Results

**Fig 1:**
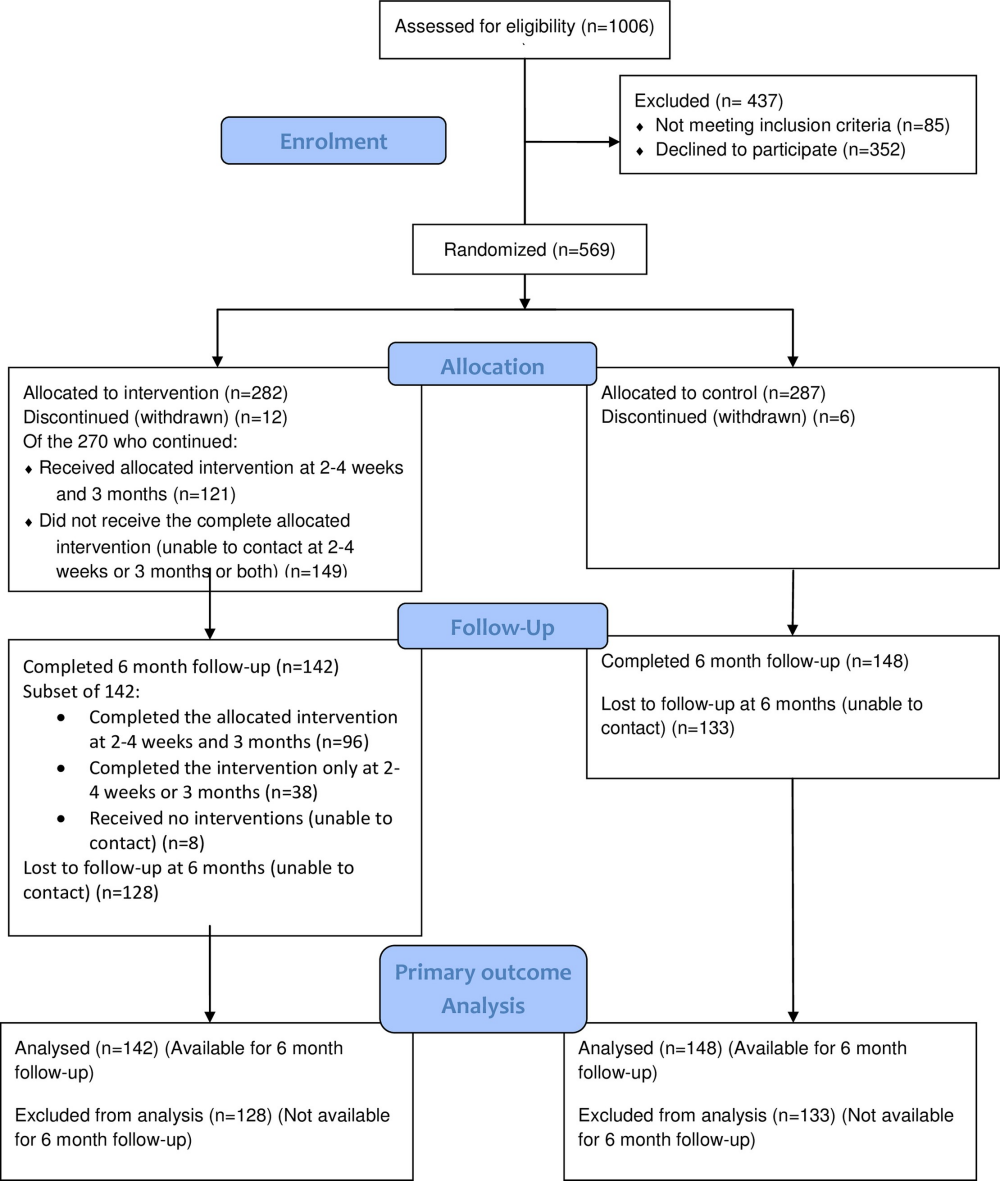
Participant flow diagram.

1006 women were assessed for eligibility; 437 were excluded; amongst these, 352 declined and the remaining 85 were not eligible. 569 patients were randomised: 282 to the intervention (I) arm and 287 to the control (C) arm. 18 patients withdrew from the study (ten decided not to have an abortion and eight did not wish to continue with the study). A total of 290 participants (I = 142, C = 148) completed the six-month follow-up (Fig 1).

Table 1 describes the cohort in the randomised groups that remained after the withdrawals. The two arms were balanced in their baseline characteristics. Almost half were Black African/Black Caribbean/Black British; the majority were educated to at least secondary level and over half in both groups were educated to tertiary level.

**Table 1:** Baseline demographics and characteristics of randomised study participants.

		Control (N = 281)	Intervention (N = 270)
**Age in years (mean, SD)**		27·7 (6·2)	26·8 (6·6)
**Ethnicity [n (%)]**	Black	145 (52)	131 (48)
	White	100(35)	110 (41)
	Asian	11 (4)	11 (4)
	Mixed/other	25 (9)	20 (7)
**Highest education attained [n (%)]**	None	0 (0)	2 (1)
	Primary	7 (2)	7 (3)
	Secondary	103 (37)	120 (44)
	Tertiary	171 (61)	141 (52)
**Current employment status [n (%)]**	Employed	125 (46)	121 (45)
	Unemployed	61 (22)	70 (26)
	Student	70 (25)	65 (24)
	Carer	22 (8)	14 (5)
**In relationship at time of abortion [n (%)]**		202 (72)	186 (69)
**Ever had a live birth [n (%)]**		151 (54)	130 (48)
**Previous abortions [n (%)]**	0	136 (48)	141 (52)
	1	98 (35)	91 (34)
	2	38 (14)	30 (11)
	3	6 (2)	5 (2)
	4	3 (1)	3 (1)
**Effective contraception method used prior to abortion [n (%)]**		156 (56)	147 (54)
**Effective contraception method planned post-abortion [n (%)]**		156 (56)	147 (54)
**Abortion referrer [n (%)]**	GP	106 (38)	122 (45)
	Sexual health/family planning clinic	4 (1)	2 (1)
	Self	97 (35)	87 (32)
	Other	74 (26)	59 (22)
**Abortion type [n (%)]**	Surgical	182 (65)	189 (70)
	Medical	99 (35)	81 (30)

Almost half were employed and had a previous live birth. Over 50% had had at least one previous termination of pregnancy. The cohort who completed the six-month follow-up showed no statistically significant differences in their baseline characteristics compared to those who did not complete the follow-up (Table 2).

**Table 2:** Attrition analysis: Comparison of baseline characteristics between those who were available for the six-month follow-up versus those who were lost-to-follow-up (FU) at six months.

		Did not complete 6-month FU (N = 261)	Completed 6-month FU (N = 290)	P value
**Age in years (mean, SD)**		26·9 (6·1)	27·5 (6·7)	0·31
**Ethnicity [n (%)]**	Black	124 (48)	152 (52)	0·55
	White	106 (41)	104 (36)	
	Asian	9 (3)	13 (4)	
	Mixed/other	22 (8)	21 (7)	
**Highest education [n (%)]**	None	1 (0·4)	1 (0·4)	0·54
	Primary	6 (2)	8 (3)	
	Secondary	114 (44)	109 (38)	
	Tertiary	140 (54)	172 (59)	
**Employment status [n (%)]**	Employed	113 (43)	136 (47)	0·51
	Unemployed	69 (26)	62 (21)	
	Student	64 (25)	71 (25)	
	Carer	15 (6)	21 (7)	
**In relationship at time of abortion [n (%)]**		181 (70)	207 (71)	0·6
**Ever had a live birth [n (%)]**		132 (51)	149 (51)	0·85
**Previous abortions [n (%)]**	0	132 (51)	145 (50)	
	1 or more	129 (49)	145 (50)	0·81
**Effective contraception method used prior to abortion [n (%)]**		143 (55)	160 (55)	0·94
**Effective contraception method planned post-abortion [n (%)]**		143 (55)	160 (55)	
**Abortion referrer [n (%)]**	GP	99 (38)	129 (45)	0·06
	Sexual health/family planning	3 (1)	3 (1)	
	Self	102 (39)	82 (28)	
	Other	57 (22)	76 (26)	
**Abortion type [n (%)]**	Surgical	174 (67)	197 (68)	0·75
	Medical	87 (33)	93 (32)	

### Primary outcome

Data from 290 patients (142 (Intervention) and 148 (Control)) who were available for the six month follow-up, were analysed for the intention-to-treat primary outcome analysis. 62% (88/142) of women in the intervention group were using effective contraception at six months post-abortion compared to 54% (80/148) in the control group, with mean difference of 8% [95% Confidence Interval CI -3·4 to 19·2%]; p = 0·172 (Table 3). Additional adjustment for centre did not change the results (p = 0·185).

**Table 3:** Primary and secondary outcome analysis between the intervention and control groups on intention-to-treat population.

	Intervention	Control	Mean difference % and [95% CI]	P value
**Primary outcome**				
**Effective contraception at six months** **[n/N (%)]**	88/142 (62%)	80/148 (54%)	8 [-3·4 to 19·2]	0·172
**Secondary outcomes**				
**1. Subsequent abortion within one year post-index abortion [n/N (%)]**	26/270 (10%)	28/281 (10%)	0·3 [-4·6 to 5·3]	0·895
**2. Subsequent abortion one to two years post-index abortion [n/N (%)]**	15/270 (6%)	16/281 (6%)	0·1 [-3·7 to 4·0]	0·944

### Secondary outcomes

Data from 551 patients (270 (Intervention), 281 (Control)) were analysed for the intention-to-treat secondary outcome analysis. Subsequent abortions within one and two years post-index abortion were similar in the intervention and control groups; subsequent abortion within one year was10%, and between one and two years was 6%. Details of these results are illustrated in Table 3.

Further exploratory analyses on the intention-to-treat population showed that the use of LARC method at six months was higher in the intervention group, but not statistically significant (42% versus 32%; p value = 0·084). A non-significant effect was observed in patients’ satisfaction with their contraceptive method used at six months in the intervention group compared to the control group (87% versus 79%; p = 0·110). Details of these results are illustrated in Table 4.

**Table 4:** Analysis of LARC use at six months post-abortion and satisfaction with chosen contraceptive method on intention-to-treat population.

	Intervention	Control	Mean difference% and [95% CI]	P value
**1. LARC**				
**LARC at six months [n/N (%)]**	60/142 (42%)	48/148 (32%)	10 [-1·3 to 20·9]	0·084
**2. Satisfaction**				
**Satisfaction with chosen contraceptive method at six months [n/N (%)]**	116/134 (87%)	111/140 (79%)	7 [-1·5 to -16·1]	0·110

### Per-protocol analyses

In the per-protocol population, 121 women received the complete intervention package but only 96 of these were available for the six-month follow-up. From the control arm, 148 women were available for the six-month follow-up. The baseline characteristics of the 244 patients included in the per-protocol analyses were also balanced (S1 Table).

Women who received the complete intervention package were significantly more likely to use effective contraception (67% versus 54%, p = 0·048) and use LARC (49% versus 32%, p = 0·010) at six months post-abortion. However, satisfaction with chosen method of contraception remained non-significant between groups. There was a reduction in subsequent abortions within one and two years post-index abortion in the group that received the complete intervention package compared to control group, although this did not reach statistical significance (subsequent abortion within one year 5% versus 10%, p = 0·098, subsequent abortions between one and two years 3% versus 6%, p = 0·164). Details of these results are illustrated in Table 5.

**Table 5:** Contraception method use, subsequent abortions and satisfaction with chosen contraceptive method between the intervention and control groups in patients who completed the intervention (per-protocol analyses).

	Intervention	Control	Mean difference % and [95% CI]	P value
**Effective contraception at six months [n/N (%)]**	64/96 (66·7%)	80/148 (54·05%)	13 [0·2 to 25·0]	0·048
**Subsequent abortion within one year post-index abortion [n/N (%)]**	6/121 (5%)	28/281 (10%)	5 [-0·2 to 10·2]	0·098
**Subsequent abortion one to two years post-index abortion [n/N (%)]**	3/121 (3%)	16/281 (6%)	3 [-0·1 to 7·1]	0·164
**LARC at six months [n/N (%)]**	47/96 (49%)	48/148 (32%)	17 [4.0 to 29.1]	0·010
**Satisfaction with chosen contraceptive method at six months [n/N (%)]**	81/92 (88%)	111/140 (79%)	8 [-0·06 to -18·2]	0·084

In the per-protocol population, a higher proportion of women had changed from using no contraception or a non-LARC method prior to abortion to a LARC method at six months (50%) compared to those in the control group (31%) (p value = 0·004). The change from non-LARC or no method prior to abortion to LARC at six months post-abortion was two-fold higher in those who completed the intervention compared to the control group (odds ratio = 2·21 [95% CI 1·28 to 3.83]). These rates were also significant in the intention-to -treat population (43% vs 31%, p-value = 0.04; odds ratio = 1.67 [95% CI 1.01 to 2·75]).

## Discussion

This study showed that a structured specialist support package following abortion did not increase the use of effective contraception at six months post-abortion. There was no significant difference in the incidence of subsequent abortions within two years between the intervention and control groups. However, participants who engaged in the interventions showed a positive effect on effective contraceptive use, in particular long-acting contraceptive methods. A reduction was observed in subsequent abortions within one and two years in the per-protocol population, but did not reach statistical significance.

A recent systematic review [15] including six RCTs had found no evidence of enhanced peri-abortion counselling on uptake of effective contraception or subsequent pregnancies; however the studies included showed variation in timing, duration and the specific nature of intervention, with some reporting evidence of effect [3,11–13]. There is limited evidence on the effectiveness of follow-up contraceptive support interventions after abortion. Studies from developing countries focusing on counselling interventions provided during the period following abortion have shown a positive effect on uptake of effective contraception [13,22].

To our knowledge, our study is the first RCT in the United Kingdom assessing a specialist contraceptive intervention provided exclusively in the post-abortion period. One of the main challenges was recruitment, typical of studies recruiting from clinics dealing with sensitive matters such as abortion [23]. The participating clinics offered same day consultation and abortion procedure, and finding the opportunity to screen patients for recruitment during the small window period between the pre-abortion consultation and the abortion procedure proved challenging. Recruitment was slower than anticipated, and two of the centres were unable to continue participation until target numbers could be reached. We did not offer incentives to participants to increase participation and retention. Our lost-to-follow-up rates of 40% at three months and 47% at six months, although comparable to previous studies [3,8,11], was a limitation of this study, decreasing the statistical power of our primary outcome assessment. It is possible that some participants in the control group may not have been available for the six month follow-up due to despondency at not being allocated to the intervention arm; Nevertheless, loss-to-follow-up at six months was similar in the control and intervention groups and baseline characteristics of our participants who responded to the six-month follow-up were similar to those who did not (Table 2). Loss of motivation, no sexual partner, not in need of or not appreciative of the services provided [24], change of telephone number, disconnected mobile phones, and an itinerant local population could be some of the reasons why women were not available for follow-up. The majority of patients requested contact through mobile phone calls and SMS text messaging. Alternative contact via e-mail or social media may have increased effective patient engagement and retention. There is also the potential for self-selection bias as the patients who agreed to participate in the study and completed the intervention may have been more motivated to receive contraceptive support. It is possible that individuals who declined to participate or dropped out of the study may have been different in regards to contraceptive use than those who stayed in. Participants were not required to disclose reasons for declining to take part in the study or what their contraceptive choices were. Among the patients who were screened, we sought to understand demographic differences between those who declined versus those who consented to participate, by submitting an amendment to our Ethics Committee five months into the study requesting permission to analyse routinely collected data on age and ethnicity. There were no statistically significant differences in age and ethnicity between the two groups (S2 Table). Additionally there is the possibility of ascertainment bias from the research nurse who was interviewing both arms of the study and was unblinded. To minimise this bias, a standardised questionnaire was used to assess contraceptive use at six months in both the control and intervention arm participants. Due to the unblinded nature of the intervention, it is possible that patients in the intervention arm could have deliberately failed to disclose ineffective use of contraception for fear of being judged; however such social desirability bias is also possible with responses from the control arm patients.

Our randomisation process ensured that the study arms were balanced in their baseline characteristics. It was stratified by centre to eliminate bias arising from differences between abortion centres regarding client characteristics, pre-abortion contraceptive counselling and immediate post-abortion contraceptive provision.

Despite the majority of our study participants reporting no plans for a pregnancy in the next 12–24 months (S3 Table), just over half reported intention to use an effective method of contraception post-abortion (Table 1). Over half of all patients in the control group reported seeking advice about contraception during the six-month period following abortion (S3 Table); those who received the full package of intervention however showed significantly higher use of effective contraception. In the absence of organised follow-up, women who do not commence an effective method on the day of abortion, or those who experience difficulties with the method chosen may ‘miss out’ on the opportunity for specialist contraceptive advice. Madden et al reported that women who attended for timely follow-up visits after their abortion were less likely to have a subsequent pregnancy within 12 months than were women who presented late [25].

Initiating contraception on the day of abortion, in particular LARC, has proven significant in the reduction of subsequent abortions [2,26]. A study by Aiken et al reported that 85% of women undergoing a termination of pregnancy from BPAS clinics in England and Wales accepted contraceptive counselling; 51% chose to obtain their method from the same provider, 33% indicated a preference to obtain their contraception from their GP or a family planning clinic, 7% did not need contraception for a range of reasons and 8% declined counselling or contraception [27]. Other studies exploring women’s desires for contraceptive counselling at the time of their abortion have reported that about one-third [28] to two-thirds [29] of women were not interested in receiving counselling on the day of abortion. Interestingly, among women using LARC at six months in our study, more women in the group that completed the intervention reported starting that method between one and six months post-abortion (30%) compared to the control group (4%), which is a likely effect of the specialist contraceptive counselling women received during this period.

It has also been reported that women who have had a recent abortion have lower rates of contraceptive continuation and a higher risk of subsequent unintended pregnancy compared to women who never had an abortion [6]. Recent qualitative research has highlighted the difficulties which young women may experience with their contraceptive method chosen at abortion, suggesting the need for long-term support to help managing side effects or switching to alternative contraception [30]. In addition, there is a natural delay in the immediate post-abortion contraceptive uptake of intrauterine contraceptive methods in women undergoing medical abortions where the second stage occurs at home [27]. As medical abortions constitute an increasing proportion of all induced abortions in England and Wales (62% of all induced abortions in 2016 versus 30% in 2006) [1], more women may be losing out on the opportunity for the full range of LARC methods in the absence of organised contraceptive follow-up. Potential benefits of contraceptive support interventions following abortions include the timing of the specialist contraceptive support package away from the stressful peri-abortion period, facilitated access to LARC initiation with help in managing side effects, and the opportunity for follow-up for women undergoing medical abortion.

Monitoring unintended pregnancies for extended periods of time is one of the most reliable indices of effectiveness of contraceptive counselling [14]. Pregnancies ending in an abortion within a short period after an abortion are likely to be unintended and may have been avoided through secondary prevention strategies. Hence we sought to investigate subsequent abortion rates within two years as a secondary outcome among our study participants. However our study was not powered to detect an effect on subsequent abortions. Schunmann et al [3] looked at the effect on subsequent abortions up to two years post-abortion using data from case note reviews. As this method carries a risk of underestimation, and patient interviews carry the potential for self-reporting bias, we linked our data with the national abortion database for two years post-abortion, which provided robust objective information. Despite the value of this method, it had a few limitations. Data on subsequent abortions could have been missed for patients who changed their address during the study period. It is possible that some of the unintended pregnancies that may have occurred shortly after the abortion could have resulted in childbirth, ectopic pregnancies or miscarriage. We did not study these outcomes hence relying on the subsequent abortion data alone could have potentially underestimated the total number of unintended pregnancies.

A larger sample size with specialist intervention targeted for a select population, for example, those who remain undecided about their contraceptive method post-abortion, and women who undergo medical abortion, may have changed the outcome of the study. However, there are real practical challenges to recruitment in the abortion setting which could make studies with large sample sizes unachievable and very expensive. To eliminate the potential dilution of any intervention effect arising from lost-to-follow-ups, future studies could use subsequent abortions through data-linkages with routinely collected national abortion datasets as the primary outcome. However such studies need much bigger sample sizes. Furthermore, national population-level reporting of the incidence of abortions within one-to-two years after an abortion could help evaluate the impact of interventions to improve post-abortion contraception.

The potential benefits of timely interventions tailored to support effective contraceptive use by women undergoing abortion may be realised through strategies which optimise patient engagement. Further research is required to identify contextual barriers to follow-up interventions to improve post-abortion contraception. Collaborative initiatives by abortion services and contraceptive specialists can help to engage patients in need of additional contraceptive support post-abortion, ensuring a seamless pathway of support.

## Supporting information

S1 Consort Checklist (DOC)

S1 Table Baseline demographics and characteristics of participants included in per-protocol analyses. (DOCX)

S2 Table Demographic comparisons between those who declined participation in the study versus those who agreed to participate (data collected from 1^st^ March 2012). (DOCX)

S3 Table Comparison of post-abortion pregnancy intentions and other characteristics in intervention and control groups, reported by participants at the 6-month interview (intention-to-treat population). (DOCX)

S1 Topic Guide Topic guide for data collection sections A, B, C, D and E.
(DOCX)

S1 Trial Protocol (DOC)
