# Supplementary material for: Specialist follow-up contraceptive support after abortion—Impact on effective contraceptive use at six months and subsequent abortions: A randomised controlled trial
Source: PLoS One. 2019 Jun 11;14(6):e0217902. doi: 10.1371/journal.pone.0217902 (PMC6559659; doi:10.1371/journal.pone.0217902)
Supplement: S1 Table — (DOCX) [file pone.0217902.s002.docx]

**S1 Table**

|  |  | Control (148) | Intervention (96) | P value |
| --- | --- | --- | --- | --- |
| Age in years (mean, SD) | Years (SD) | 27·6 (6·6) | 28·1 (7·1) | 0·56 |
| Ethnicity [n (%)] | Black | 88 (60) | 46 (48) | 0·28 |
|  | White | 44 (30) | 37 (39) |  |
|  | Asian | 5 (3) | 6 (6) |  |
|  | Mixed/other | 11 (7) | 7 (7) |  |
| Highest education attained [n (%)] | None | 0 (0) | 1 (1) | 0·24 |
|  | Primary | 5 (3) | 3 (3) |  |
|  | Secondary | 47 (32) | 40 (42) |  |
|  | Tertiary | 96 (65) | 52 (54) |  |
| Current employment status [n (%)] | Employed | 70 (47) | 42 (44) | 0·53 |
|  | Unemployed | 28 (19) | 25 (26) |  |
|  | Student | 38 (26) | 24 (25) |  |
|  | Carer | 12 (8) | 5 (5) |  |
| In relationship at time of abortion [n (%)] |  | 104 (70) | 68 (71) | 0·93 |
| Ever had a live birth [n (%)] | Yes | 81 (55) | 48 (50) | 0·47 |
| Previous abortions [n (%)] | 0 | 72 (49) | 47 (49) | 0·95 |
|  | 1 | 56 (38) | 39 (41) |  |
|  | 2 | 15 (10) | 7 (7) |  |
|  | 3 | 2 (1) | 1 (1) |  |
|  | 4 | 3 (2) | 2 (2) |  |
| Effective contraceptive method used prior to abortion  [n (%)] | | 82 (55) | 53 (55) | 0·98 |
| Effective contraceptive method planned post- abortion  [n (%)] | | 82 (55) | 53 (55) | 0·98 |
| Abortion referrer [n (%)] | GP | 60 (41) | 50 (52) | 0·18 |
|  | Sexual health/family  planning clinic | 45 (31) | 18 (19) |  |
|  | Self | 41 (28) | 27 (28) |  |
|  | Other | 2 (1) | 1 (1) |  |
| Abortion type [n (%)] | Surgical  Medical | 96 (65)  52 (35) | 70 (73)  26 (27) | 0·19 |
