# Supplementary material for: Specialist follow-up contraceptive support after abortion—Impact on effective contraceptive use at six months and subsequent abortions: A randomised controlled trial
Source: PLoS One. 2019 Jun 11;14(6):e0217902. doi: 10.1371/journal.pone.0217902 (PMC6559659; doi:10.1371/journal.pone.0217902)
Supplement: S2 Table — (DOCX) [file pone.0217902.s003.docx]

**S2 Table**

|  |  | Enrolled  (337) | Declined (136) | p-value |
| --- | --- | --- | --- | --- |
| Mean age (SD) |  | 27.2 (6.2) | 27.8 (7.1) | 0.32 |
| Median age (IQR) |  | 27 (22, 31) | 26 (23, 32) | 0.66 |
| Ethnicity* | **white** | 122 (37) | 37 (30) | 0.15 |
|  | **black** | 173 (52) | 66 (53) |  |
|  | **Asian/other** | 15 (5) | 12 (10) |  |
|  | **mixed** | 24 (9) | 9 (7) |  |

*Ethnicity data was not available for 3% of screened participants
