# Supplementary material for: Specialist follow-up contraceptive support after abortion—Impact on effective contraceptive use at six months and subsequent abortions: A randomised controlled trial
Source: PLoS One. 2019 Jun 11;14(6):e0217902. doi: 10.1371/journal.pone.0217902 (PMC6559659; doi:10.1371/journal.pone.0217902)
Supplement: S3 Table — (DOCX) [file pone.0217902.s004.docx]

**S3 Table**

|  |  | Control (n=148) | Intervention (n=142) | P value |
| --- | --- | --- | --- | --- |
| Contact with healthcare professional for contraceptive advice since recent abortion (n,%) |  | 80 (54) | 90 (63) | 0·11 |
| Pregnancy plan at one year (n,%) | Yes | 15 (10) | 8 (6) | 0·16 |
|  | No | 111 (76) | 115 (81) | 0.22 |
|  | Not sure | 21 (14) | 19 (13) | 0.84 |
| What would you do if you accidently fell pregnant? (n,%) | Continue pregnancy | 68 (46) | 55 (39) | 0·21 |
|  | TOP | 16 (11) | 22 (15) | 0.24 |
|  | Assured never happen again | 6 (4) | 1 (1) | 0.06 |
|  | Not sure | 57 (39) | 64 (45) | 0.26 |
| Unprotected sexual intercourse during the 6 months post-abortion (n,%) |  | 28 (19) | 19 (13) | 0·20 |
| New partner since abortion (n,%) |  | 16 (11) | 20 (14) | 0·40 |
| Pregnancy plan at one-to-two years (n, %) | Yes | 39 (27) | 31 (22) | 0·37 |
|  | No | 84 (57) | 82 (58) | 0.87 |
|  | Not sure | 24 (16) | 29 (20) | 0.35 |
