# Supplementary material for: Specialist follow-up contraceptive support after abortion—Impact on effective contraceptive use at six months and subsequent abortions: A randomised controlled trial
Source: PLoS One. 2019 Jun 11;14(6):e0217902. doi: 10.1371/journal.pone.0217902 (PMC6559659; doi:10.1371/journal.pone.0217902)
Supplement: S1 Topic Guide — Topic guide for data collection sections A, B, C, D and E. (DOCX) [file pone.0217902.s005.docx]

**S1 Topic guide**

1. Baseline data collection at recruitment: (for both the ‘Control’ and ‘Intervention’ groups)

- Date of Birth
- Ethnicity
- Post code
- Educational achievement
- Employment status
- Marital/Relationship status
- Parity
- Previous abortions
- Contraceptive use immediately prior to current pregnancy
- Planned method of contraception after abortion
- Who referred for the abortion : Self/GP/Family Planning or Sexual Health clinic/other

1. Data collection at the two-to-four week consultation with the Specialist in Reproductive and Sexual Health: (This was for the ‘Intervention’ Group only)

- Any change in the relationship status
- Whether accompanied by partner for this visit (if having a face-face consultation in a clinic)
- Smoking
- Alcohol use
- Drug misuse
- Domestic violence
- Relevant Medical History
- Any physical problems following the abortion
- Level of psychological distress following abortion
- Any additional psychological support required
- Any concerns about future fertility
- Any unprotected sexual intercourse since the last abortion
- Any use of Emergency contraception since the last abortion
- Contraceptive method provided at the time of abortion
- Current method of contraception
- When was the method started
- Is the contraceptive method being effectively used
- Any side effects from the contraceptive method
- Any particular method of contraception medically contraindicated
- Previous contraceptive use
- Any problems with previous contraceptive methods
- Any particular contraceptive method/s unacceptable to the patient
- Attitude to future unplanned pregnancy
- Awareness of safer sex practice, STIs
- Contraceptive method provided at this visit
- Arrangements for follow-up
- Any referral to other agencies/other healthcare professionals:
- Social services
- Safeguarding children team
- Safeguarding adults team
- Domestic violence support
- Alcohol and Drug misuse
- Mental Health team
- Psychologist
- Health advisor/counsellor
- Haven (sexual assault services)
- Services for trafficked women
- Support services for women working in prostitution
- General Practitioner

1. Phone call to study participants who DNA the two-to-four week appointment (Intervention group only)

- Reason for DNA
- Offer suitable alternative appointment (telephone consultation or face-to-face clinic appointment) as preferred by the patient
- Telephone assessment (as in Section B) if willing
- Telephone Intervention purely focused on facilitating contraceptive uptake if not willing for all the details in Section B
- Link with other healthcare professional as necessary

1. Phone call to study participants at three months post-abortion (Intervention group only)

- Change in the relationship status
- Contact with GP or contraception service for contraceptive advice between the two-week appointment and now
- Current method of contraception
- When was the method started
- Is the contraceptive method being effectively used
- Any side effects from the contraceptive method
- Any unprotected sexual intercourse since the recent abortion
- Any use of Emergency contraception since the recent abortion
- Any further support required
- Arrangements for follow-up

1. Phone call to study participants six months post-abortion ( Intervention and Control groups)

- Any change in the relationship status
- Current method of contraception
- When was the contraceptive method started
- Is the contraceptive method being effectively used
- Patient satisfaction with the contraceptive method
- Any unprotected sexual intercourse since the recent abortion
- Any use of Emergency contraception since the last abortion
- Any contact with GP/contraception service for contraceptive advice between the abortion and now
- Pregnancy intentions for the next one and two years
- Attitude to future unplanned pregnancy
- Alcohol use
- Drug misuse
- Domestic (Intimate partner) violence
