# Supplementary material for: Specialist follow-up contraceptive support after abortion—Impact on effective contraceptive use at six months and subsequent abortions: A randomised controlled trial
Source: PLoS One. 2019 Jun 11;14(6):e0217902. doi: 10.1371/journal.pone.0217902 (PMC6559659; doi:10.1371/journal.pone.0217902)
Supplement: S1 Trial Protocol — (DOC) [file pone.0217902.s006.doc]

# PROTOCOL TITLE:

| **Randomised controlled study of the impact of provision of follow-up contraceptive support to women who have had an abortion.** |
| --- |

### Trial Identifiers

| REC Ref.Number | 11/H0709/1 |
| --- | --- |

### (Co) Sponsor(s)

| Name: King’s College Hospital NHS Foundation Trust  Address: Denmark Hill, London SE5 9RS  Telephone: 02032999000  Fax: 02032995515  Email: kch-tr.research@nhs.net |
| --- |

### Chief Investigator

| Name: Dr.Usha Kumar  Address: Camberwell Sexual Health Centre, King’s College Hospital NHS Foundation Trust, 100 Denmark Hill, London SE5 9RS  Telephone: 0208 3299 5043  Fax: 0207 733 2477  Email: u.kumar@nhs.net |
| --- |

### Name and address of Co-Investigator(s), Statistician, Laboratories etc

| Name: Dr.Susan Mann  Address: Camberwell Sexual Health Centre, King’s College Hospital NHS Foundation Trust, 100 Denmark Hill, London SE5 9RS  Telephone: 07980644325  Fax: 0207 733 2477  Email: suemann1@nhs.net  Name: Dr.Paula Baraitser  Address: Camberwell Sexual Health Centre, King’s College Hospital NHS Foundation Trust, 100 Denmark Hill, London SE5 9RS  Telephone: 07525630865  Fax: 0207 733 2477  Email:Paula.baraitser@nhs.net  Name: Dr.Abdel Douiri  Address: Primary Care and Public Health Sciences, King’s College London, Capital House, Floor 5, Room 505, 42 Weston Street, London SE1 3QD  Telephone: 02078488224  Fax:  Email: abdel.douiri@kcl.ac.uk |
| --- |

### CONTENTS

1.Background & Rationale 4-7

2 Research Objectives and Design…………………………………………………………………………………. 8-11

2.1. Research Objectives 8

2.2 Research Design………………………………………………………………………………………………………….8-9

2.3 Research Flowchart 9-11

3. Selection and Withdrawal of Subjects 11-12

3.1 Inclusion Criteria 11

3.2 Exclusion Criteria 11

3.3 Selection of Participants 11

3.4 Randomisation Procedure 12

3.5 Withdrawal of Subjects 12

3.6 Expected Duration of Trial. 12

4. Study Procedures 12-14

4.1Patient Information……………………………………………………………………………………………………….12

4.2Screening and Eligibility 12

4.3Baseline assessment 12

4.4Informed Consent………………………………………………………………………………………………………….13

4.5Randomisation…………………………………………………………………………………………………………………….13

4.6 Intervention…………………………………………………………………………………………………….………13-14

4.7 Study Endpoints…………………………………………………………………………………………………………………14

5. Ethics………………………………………………………………………………………………………………………… 15

5.1.Consent………………………………………………………………………………..............................................15

5.2 Confidentiality……………………………………………………………………………………………………………..15

5.3 Vulnerable clients……………………………………………………………………………………………………………….15

6 Statistics………………………………………………………………………………........................................16-17

6.1 Sample Size…………………………………………………………………………......................................15-16

6.2 Randomisation 16

6.3 Analysis 16

7. Data Monitoring Committee…………………………………………………………………………………………17

8. Direct Access to Source Data and Documents 17

9. Ethics & Regulatory Approvals 17

10. Quality Assurance 17

11. Data Handling……………………………………………………………………………………………………………….18

12. Publication Policy 18

13. Financial Aspects 18

14. Signatures 18

15. Appendices…………………………………………………………………………………………………………………...19-30

15.1 Appendix 1 Patient information sheet and consent form……………………………………..……..20-25

15.2 Appendix 2 Topic Guides for data collection ……………………………………………………………….26-30

| 1. Background & Rationale In 2009, 189,100 abortions were carried out for women resident in England & Wales. 34% of these women had one or more previous abortions, an increase from 29% since 1998. The boroughs of Lambeth, Southwark and Lewisham have some of the highest rates of unplanned pregnancies and abortions in the UK. In 2009, there were 6,747 abortions amongst residents of Lambeth, Southwark and Lewisham with a rate of 34/1000 resident women between the ages of 15- 44 [1].This is nearly twice the national average abortion rate. 39% of these were repeat abortions.  The increase in repeat abortions is despite the widespread availability of contraceptive and emergency contraceptive services. This is a reflection of poor uptake and continuation of effective contraception post-abortion and highlights the gap between provision of abortion care and effective post-abortion care.  Reducing the number of repeat abortions remains a big challenge for the NHS. Several studies have attempted to investigate risk factors for repeat abortions to identify the high risk groups for targeted interventions [2-10]. Risk factors include young age, poor socio-economic status, unemployment, poor educational background, parous women, single/cohabiting with boyfriend, divorced/ separated/widowed women, multiple sexual partners, early age at sexual debut, unwanted sex, history of physical/sexual abuse, intimate partner violence, difficult schooling, defective parental images, geographic or social instability, psychological factors such as low self esteem, passivity, dependence, immaturity, depressive personality, previous psychiatric treatment, unstable relationships, failure to use contraception, associated with ambivalence in relation to desire for children or moral aversion to the use of contraception, non-attendance at follow up visits post-abortion, and non-participation of male partners in abortion decision-making.  Pregnancies ending in repeat abortions within a short time-frame are likely to be unintended pregnancies that might have been avoided. Abortion itself provides an opportunity for secondary prevention. The RCOG Guideline on abortion care recommends the provision of contraceptive advice as part of abortion counselling and the provision of a contraceptive method according to the woman’s choice to start straightaway after the abortion [11].  The London Sexual Health Strategic Framework has defined standards and outcome indicators for improving the Sexual Health of Londoners [12]. This includes the need to improve care pathways to promote post-abortion contraception with the aim of reducing repeat abortions. In accordance with this all abortion providers should be commissioned to provide contraception including Long acting methods of contraception (LARC) and all women leaving the service should be provided with one of the most effective methods of contraception (oral contraceptive pill, injection, Implant or Intrauterine device). Most of the abortion care in the NHS is contracted to the independent sector or provided by Hospital Trusts neither of whom hold ongoing responsibility for provision of continuing contraceptive care creating a disjunction between the two.  Previous studies have shown that contraceptive risk-taking is high both before and after abortion and that peri-abortion contraceptive counselling was ineffective or inadequate [13, 14]. A more thorough, client-centred & focussed peri-abortion contraceptive counselling with structured follow-up has been suggested by previously published research [15,16,17]. An audit of peri-abortion contraceptive counselling practices in a UK hospital showed that the content of contraceptive advice given to women undergoing abortion varied with only 40% receiving a comprehensive discussion on contraceptive options in relation to individual circumstances, and follow up had been arranged for less than half of the women [16].  A qualitative study of user experience of abortion services in Lambeth, Southwark & Lewisham highlighted the ineffectiveness of the system of peri-abortion contraceptive counseling, where there was no clarity about the roles of various service providers in provision of detailed and effective contraceptive counselling and follow-up, with a tendency for healthcare professionals to assume that the next person along the line would deal with contraception [14]. There was over-reliance on the ad-hoc post abortion follow up visit which the clients were expected to organize themselves.  Effective contraceptive counseling is time consuming especially for women who have already undergone an abortion as their contraceptive needs may be complex due to the various psycho-social aspects involved. It may be impractical to provide such counseling within the time available to staff during the referral visit, at the pre-abortion assessment or on the day of abortion. Moreover, women may not be in the right frame of mind to absorb all the information about contraception and make informed decisions during a stressful period.  A review of women presenting for repeat abortion within 24 months of the first abortion showed that although 58% accepted LARC following abortion, only 2% continued its use thereafter and 50% of women were not using any contraception at the time of abortion. Contraceptive counseling that educates them regarding expected side-effects of LARC and follow-up could ensure better compliance [18].  A recent study on abortion and repeat abortion in young women from London recommended the need for follow-up appointments for all young women undergoing abortion, which would address the whole woman and include intensive and individualized counseling based on their needs [19].  Focussing post-abortion care in a dedicated Specialty clinic providing contraceptive counseling has been shown to improve contraceptive uptake and reduce the incidence of repeat pregnancies [20].  Interventional studies that involved provision of a comprehensive specialist individualised contraceptive counseling & provision to women undergoing abortions have shown a better uptake of effective methods of contraception as compared to a package of limited information [21- 23]. Randomised studies of such interventions are scarce [21,23]. Specialist contraceptive advice on the day of abortion and enhanced provision had a short-lived effect on contraceptive uptake and increased the use of long-acting methods of contraception but did not appear to reduce repeat abortions [21].  A cohort study of women who underwent first trimester surgical abortions at a hospital based clinic in New York reported that women who returned for timely follow-up visits post-abortion were less likely to have subsequent repeat pregnancy and abortion in the 12 months post-abortion [24].  Lambeth, Southwark and Lewisham (LSL) jointly commission abortion services from 3 providers, King’s College Hospital NHS Trust, Marie Stopes International (MSI) and British Pregnancy Advisory service (BPAS). Provision of post-abortion contraception is included in the commissioning of abortion services. Specific structured follow-up for patients after the abortion to support contraceptive decision-making or contraceptive continuation is not routinely organized when women present for an abortion. Patients are advised to see their GP or referring doctor after 2 weeks for their routine follow-up. Patients may not be motivated to arrange such follow up if they have no problems related to the abortion itself.  In an effort to reduce the high rate of repeat abortions among young women in Lambeth, Southwark & Lewisham, there have been recent local pilot initiatives to try and follow up young women after their abortion to address their contraceptive needs. Evaluations of such an initiative from Lewisham providing follow up support to young women under the age of 18 during the period June 2008-June 2009, reported an impressive effect on uptake of reliable contraception post abortion, with the percentage of women relying on condoms alone following abortion falling to 17% as compared to more than 80% prior to abortion. Recommendations have been made by local Sexual Health Commissioners for follow up of young women under the age of 25 referred for abortion from Brook services and follow up of under 19 year old’s who have had more than 1 previous abortion or leaving the abortion provider without a method of contraception.  The aim of this research is to compare the effect of a specific intervention to provide contraceptive support following abortion with that of the existing arrangement of ad-hoc follow-up. The intervention will be in the form of a structured follow-up consultation either on telephone or face-face appointment as preferred by the woman with a Specialist in a Reproductive and Sexual Health service 2-4 weeks after the abortion with telephone follow-up 3 months after to provide further contraceptive support.  It is hypothesised that such a structured follow-up arrangement with the involvement of senior staff is more likely to result in better uptake and continuation of effective contraception as they would be able to unravel complex psycho-social factors that affect motivation and decision making regarding contraception. The follow-up consultations at 2-4 weeks and again at 3 months will offer the opportunity for women to discuss any issues or side-effects they may have in relation to their chosen contraceptive method. Women in the intervention group will be encouraged to bring along their male partners during the follow-up visit if they wished, as partners may play an important role in some cases in influencing motivation, contraceptive choice, compliance and continuation. Several previous studies have highlighted the benefits of involving male partners in post-abortion contraception counselling [25-29].  It is hoped that the findings from this study will have potential implications for policy decisions regarding meaningful  follow-up interventions to reduce repeat abortions. The economic costs of unplanned pregnancy are very high comparedwith the costs of prevention through effective provision and use of contraception [30]. If effective interventions tailored to the particular needs of this group can be determined and provided there is a potential for considerable savings to be made.  **References:**   1. Abortion Statistics, England and Wales: 2009 www.dh.gov.uk/publichealthstatistics 2. Heikinheimo O, Gissler M, Suhonen S. Age, parity, history of abortion and contraceptive choices affect the risk of   repeat abortion. Contraception. 2008 Aug; 78(2): 149-54.   1. Xu JS. Huang YM. Cheng LN. Factors in relation to repeated abortions among unmarried young people in Shanghai.   Chinese Journal of Epidemiology. 28(8):742-5, 2007 Aug.   1. Cheng YM. Wang XY. Lv YH. Cai YM. Li Y. et al. Study on the risk factors of repeated abortion among unmarried   adolescents. Chinese Journal of Epidemiology. 27(8):669-72, 2006 Aug.   1. Fisher WA. Singh SS. Shuper PA. Carey M. Otchet F. et al. Characteristics of women undergoing repeat induced   abortion. CMAJ Canadian Medical Association Journal. 172(5):637-41, 2005 Mar 1.   1. St John H. Critchley H. Glasier A. Can we identify women at risk of more than one termination of pregnancy?   Contraception. 71(1):31-4, 2005 Jan.  7. Addor V. Narring F. Michaud PA. Abortion trends 1990-1999 in a Swiss region and determinants of abortion  recurrence. Swiss Medical Weekly. 133(15-16):219-26, 2003 Apr 19.  8. Kitamura T. Toda MA. Shima S. Sugawara M. Single and repeated elective abortions in Japan: a psychosocial  study.Journal of Psychosomatic Obstetrics & Gynecology. 19(3):126-34, 1998 Sep.  9. Mattauer B. Peyrot D. Aussiloux MT. Repeated requests for termination of pregnancy. Some socio-cultural and  psychological aspects. Contraception, Fertilite, Sexualite. 12(4):573-80, 1984 Apr.  10. Blumenfield M. Psychological factors involved in request for elective abortion. Journal of Clinical Psychiatry.  39(1):17-25, 1978 Jan.  11. Royal College of Obstetricians and Gynaecologists (RCOG). Care of the Woman Requesting Induced Abortion.  London: RCOG, 2004  12. The London Sexual Health Strategic Framework, 2010, London Sexual Health Programme, London Specialised  commissioning Group  13. Palanivelu LM, Oswal A. Contraceptive practices in women with repeat termination of pregnancies.  J Obstet Gynaecol. 2007 Nov;27(8):832-4.  14. Kumar U. Baraitser P. Morton S. Massil H. Peri-abortion contraception: a qualitative study of users' experiences.  Journal of Family Planning & Reproductive Health Care. 30(1):55-6, 2004 Jan.  15. David PH. Reichenbach L. Savelieva I. Vartapetova N. Potemkina R.Women's reproductive health needs in Russia:  what can we learn from an intervention to improve post-abortion care? Health Policy & Planning. 22(2):83-94, 2007  Mar.  16. Garg M; Singh M; Mansour D Peri-abortion contraceptive care: can we reduce the incidence of repeat abortions?  Journal of Family Planning and Reproductive Healthcare 2001 Apr;27(2):77-80.  17. Osler M. Morgall JM. Jensen B. Osler M. Repeat abortion in Denmark. Danish Medical Bulletin. 39(1):89-91, 1992  Feb.  18. Das S, Adegbenro A. Ray S. Amu O. Repeat Abortion: Facts and issues.Journal of Family Planning & Reproductive  Health Care. 35 (2) : 93-5, 2009 Apr.  19. Lesley Hogart and Joan Philips. Policy Studies Institute. Government office for London. Dept. for children, Schools  and families. Young people in London: Abortion and repeat abortion. Research report  http://www.younglondonmatters.org/uploads/documents/tpyoungpeopleinlondonabortionandrepeatabortion.pdf  20. Masch R. Cabrera I. Adder R. et al The effect of consolidation of abortion services on patient outcomes.  Contraception. 77(1):60-3, 2008 Jan  21. Schunmann C. Glasier A. Specialist contraceptive counselling and provision after termination of pregnancy improves  uptake of long-acting methods but does not prevent repeat abortion: a randomized trial. Human Reproduction.  21(9):2296-303, 2006 Sep.  22. Johnson BR. Ndhlovu S. Farr SL. Chipato T. Reducing unplanned pregnancy and abortion in Zimbabwe through  postabortion contraception. Studies in Family Planning. 33(2):195-202, 2002 Jun.  23. Zhu JL. Zhang WH, Cheng Y. Xu J. Xu X. Gibson D. et al The Impact of post-abortion family planning services on  contraceptive use and abortion rate among young women in China : a cluster randomised trial. European Journal of . Contraception and Reproductive Health Care. 14 (1):46-54, 2009 Feb.   1. Madden T. Westhoff C. Rates of follow-up and repeat pregnancy in the 12 months after first trimester induced abortion. Obstetrics and Gynaecology. 113 (3): 663-8, 2009 Mar. 2. Becker S. Bazant ES. Meyers C. Couples counseling at an abortion clinic: a pilot study.   Contraception. 78(5):424-31, 2008 Nov.  26. Beenhakker B. Becker S. Hires S. Molano Di Targiana N. et al. Are partners available for post-abortion contraceptive . counseling? A pilot study in a Baltimore City clinic.Contraception. 69(5):419-23, 2004 May.  27. Holmgren K. Repeat abortion and contraceptive use. Report from an Interview Study in Stockholm.  Gynecologic & Obstetric Investigation. 37(4):254-9, 1994.  28. Blumenfield M. Psychological factors involved in request for elective abortion. Journal of Clinical Psychiatry.  39(1):17-25, 1978 Jan.  29. Luker K. Understanding the risk-taker. The Family Planner. 8(5-6):10-3, 1977.  30. McGuire A, Hughes D. The Economics of Family Planning Services; A report prepared for the contraceptive  alliance. Centre for Reviews and Dissemination, York 2008. |
| --- |

### 2 Research Objectives and Design

### 2.1. Research Objectives

| The objective of the study is to assess the impact of structured specialist follow-up after abortion to provide support to patients for contraceptive decision-making and continuation on the uptake and continuation of effective contraception at 6 months and the effect on repeat abortion at 1 and 2 years. |
| --- |

### 2.2 Research Design

| This will be a randomized controlled study with an intervention arm and a control arm.  **Control arm:** Patients who have undergone an abortion under the NHS in Lambeth, Southwark and Lewisham (LSL) who will be recipients of current standard follow-up practice post-abortion, which is routine advice to see their G.P. or referring clinician 2 weeks post-abortion.  **Intervention arm**: Patients who have undergone an abortion under the NHS in LSL who will receive a specific follow up consultation with a Specialist from the Reproductive and Sexual Health Service at King’s College Hospital either by telephone or a face-face clinic appointment depending on patient preference at 2-4 weeks post-abortion to discuss contraception and a telephone follow up 3 months post abortion to provide further contraceptive support. Women will be offered the opportunity to bring their male partners to the follow-up appointment if they wished, for participation in contraceptive counseling.  Any other follow up interventions aimed at young people outside of our research intervention that may happen as a result of local commissioning recommendations in LSL will apply to participants in both the control and intervention arms. Hence any biases related to such interventions will be attenuated by the randomization design of the study.  Verbal consent to send an information leaflet about the research study will be taken by the Central Termination of Pregnancy (TOP) Booking Service when patients phone for their abortion appointment. Written patient information about the research study will be sent to potential participants along with the details of their TOP consultation appointment. Patients who do not want any documentation posted to their home address, will be approached at their pre-abortion consultation visit by a trained member of staff and offered a patient information sheet and invited to take part in the study.  Written consent for participation in the study will be obtained from patients when they attend for their TOP consultation appointment. Study participants will be randomized to the intervention or control arm by pre-determined randomization codes for each centre (strata). The Randomisation codes will be held by the Chief Investigator based in the Reproductive and Sexual Health Service at King’s College Hospital.  Patients randomized to the intervention arm will be contacted by phone by the Research nurse/fellow during the week after their TOP procedure and offered the choice of a telephone consultation or a face-face clinic appointment with a Specialist in the Reproductive and Sexual Health service at King’s College Hospital 2-4 weeks post-abortion to provide contraceptive support. For patients who choose to have a telephone consultation, a suitable time slot for the consultation will be agreed and will receive their Specialist consultation by telephone during this time. For patients who prefer a face-face consultation, a suitable appointment will be arranged to see the Specialist 2-4 weeks post-abortion. Patients in the intervention arm will also be informed about the telephone follow-up at 3 months and 6 months post-abortion for contraceptive support.  Patients randomized to the control arm will be contacted by phone by the Research nurse/fellow only at 6 months to assess contraceptive uptake and continuation.  **Primary outcome measures**  Uptake and continuation of effective contraception at 6 months. Effective contraception will be defined as regular and consistent use of oral contraceptive pills/patches/vaginal ring (Nuvaring®)/ injectable contraception/ intrauterine contraception or the contraceptive implant. Use of barrier contraception or other methods such as withdrawal methods or natural family planning will not be considered as effective contraception due to their higher relative failure rates.  **Secondary outcomes**  Repeat abortion at 1 and 2 years. |
| --- |

### 2.3 Research Flowchart

**Patient Information Sheet sent by Central TOP Booking Service when patients ring for their TOP appointment.**

**Screening and Recruitment at the TOP Provider Unit when patients attend for their TOP consultation + Baseline Data collection with questionnaire at recruitment**

**Randomised to Intervention and Control Arms**

**Intervention**

**Control**

**Research nurse/fellow will contact patients in the intervention arm by telephone during the week following their abortion procedure to arrange appointment for the 2-4 week follow up consultation (telephone/face-face depending on the patients’ preference) with the Specialist in Reproductive and Sexual Health**

**and also inform them about the telephone follow-up at 3 months and 6 months**

**2 – 4 weeks post abortion follow-up consultation for contraceptive support by telephone/face-face clinic visit with Reproductive and Sexual Health Specialist (history, physical examination +/-, STI tests +/-, treatment +/-, contraceptive provision +/-), follow up arrangements as required**

**3 months post abortion telephone interview by Research Nurse/Fellow and contraceptive support and follow up arranged as required**

**6 months post abortion telephone interview by Research Nurse/Fellow with questionnaire to assess contraceptive uptake, compliance and continuation**

**6 months post abortion telephone interview by Research Nurse/Fellow with questionnaire to assess contraceptive uptake, compliance and continuation**

**Data Linkage with Lambeth Public Health data warehouse +/- National Abortion Data from DH: repeat abortion rate 1 and 2 years after abortion .**

**Data Linkage with Lambeth Public Health data warehouse +/- National Abortion Data from DH: repeat abortion rate 1 and 2 years after abortion**

**Data Linkage with Lambeth Public Health data warehouse +/- National Abortion Data from Dept. of Health: repeat abortion rate 1 and 2 years after abortion.**

**Data Linkage with Lambeth Public Health data warehouse +/- National Abortion Data from Dept. of Health: repeat abortion rate 1 and 2 years after abortion .**

### 3 Selection and Withdrawal of Subjects

### 3.1 Inclusion Criteria

| Patients undergoing abortion under the NHS in Lambeth, Southwark and Lewisham who have consented for participation in the study. |
| --- |

### 3.2 Exclusion Criteria

| - Patients who do not speak English - Patients who will be leaving the country for 6 months after the abortion and hence will not be available for the follow up appointments or telephone follow-ups. - Patients who lack capacity to consent for themselves - Patients who attend for the pre-abortion consultation but do not go ahead with the abortion |
| --- |

### 3.3 Selection of Participants

| Patients undergoing induced abortion (any gestation, all ages) under the NHS in Lambeth, Southwark or Lewisham with one of the three abortion providers - King’s College Hospital NHS Foundation Trust, British Pregnancy Advisory Service and Marie Stopes International, who have consented to participate in the study. |
| --- |

### 3.4 Randomisation Procedure / Code Break

The design for this study will be that of a stratified block randomisation.

### Participants will be randomised by predetermined codes for each centre (strata) to intervention or control arm. The Randomisation list will be prepared by the Research statistician with a computer generated random number list stratified by centre and with random block sizes. The randomisation schedule will be incorporated into a dedicated electronic database and kept securely within this electronic system, inaccessible to the enrolment location. The allocation sequence will be concealed from the person enrolling and assessing participants to the study. The consent forms will be sent to the Chief Investigator based at King’s College Hospital at the end of each week. Participants will be assigned to the intervention or control arms based on the randomisation codes embedded within the dedicated electronic database system.

### 3.5 Withdrawal of Subjects

### Patients will be free to withdraw from the study anytime they wish. Baseline data collected on these subjects will be used for the analysis.

### 3.6 Expected Duration of Trial.

| Expected clinical participation of patients in the study will be for a period of 6 months. The total duration of the study will be 2-3 years to complete analysis of the secondary outcome of repeat abortions at 2 years. |
| --- |

**4. Study Procedures**

**4.1** Patient Information (Appendix 1):

Patient Information Sheet will be sent by Central TOP Booking Service when patients ring for their abortion appointment, after obtaining verbal consent for receiving information by post. Patients who decline postal contact will be given the information sheet when they attend for their abortion consultation.

**4.2**  Screening and eligibility:

Screening and Recruitment will be done by a member of the research team or a member of staff from the TOP Provider Unit when patients attend for their abortion consultation, after their abortion consultation.

**4.3** Baseline Assessment:

Patients approached for participation about the study will be requested to fill in a questionnaire (Appendix 2: Topic Guide A) to collect baseline socio-demographic and other relevant data relating to their sexual, reproductive and contraceptive history.

4.3(a) Retrospective data collection

Following minor amendment to the protocol (approved by NRES on 7th June 2013) we plan to collect retrospective data on abortions within the 2 years prior to recruitment in study participants. This data will allow us to better understand the contraceptive trajectories and potential future risk of repeat abortion in our recruited cohort. We will be obtaining this data through data linkage with the National abortion dataset held at the Department of Health.

**4.4** Informed Consent:

Written consent on a Consent form (Appendix 1) will be obtained from patients who are willing to participate in the study.

**4.5** Randomisation:

Patients will be randomised by predetermined codes for each centre (strata) to the intervention arm and control arm. The Randomisation list will be prepared by the Research statistician with a computer generated random number list stratified by centre and with random block sizes and held in a dedicated electronic database. The randomisation schedule will be inaccessible to the enrolment location. Patients will be allocated to the Intervention or Control arms based on the randomisation codes for each centre.

**4.6** Intervention:

1) Follow up consultation by telephone/face-face clinic appointment as per the patient’s preference with a Specialist from the Reproductive & Sexual Health Service at King’s College Hospital 2-4 weeks post-abortion. Women will be offered the opportunity to bring their male partners to the follow-up appointment if they wished, for participation in contraceptive counseling.

Further data will be collected during this follow-up consultation using a standardized questionnaire (Appendix 2 : Topic Guide B) to explore psycho-social, personal, partner-related and medical factors that could potentially influence contraceptive decision-making, compliance and continuation. The study participants will receive individualized contraceptive counseling & support and will be provided with a reliable contraceptive method of their choice if they are not already using one. Where issues such as alcohol/drug abuse, intimate partner violence, sexual exploitation, child protection, psychiatric disorders, significant psychological distress are identified, onward referral to appropriate agencies for investigation and support will be arranged. Where patients are not able to decide on a contraception plan, further follow-up appointments will be arranged as necessary to allow sufficient time for decision-making. If the patient prefers to see their GP/local contraceptive provider, a written referral will be made to the appropriate Health Care professional.

Patients will be given a telephone number and e-mail contact to contact the Chief Investigator/Research Nurse/Research Fellow if they need any additional support related to their contraception after their follow-up visit.

Management of patients who Do Not Attend (DNA) the follow-up appointment:

These patients will be contacted by telephone to identify the reason for the DNA (Appendix 2 : Topic guide C) and will be offered another suitable appointment for a telephone consultation or face-face clinic appointment with the Specialist according to the patient’s preference.

1. Telephone call 3 months post abortion to provide contraceptive support.

The Research Nurse/Fellow will contact the study participants in the intervention arm 3 months post-abortion by telephone and will carry out an interview with a standardized questionnaire (Appendix 2: Topic guide D) aimed at further data collection to include any changes in socio-demographic characteristics, personal, relationship, psychological, medical factors since the abortion and to capture information about any side-effects and compliance with the chosen contraceptive method. The Research Nurse/Fellow will also offer contraceptive support and refer back to the Specialist where necessary.

- 1. Study end points
- **Uptake and continuation of effective contraception at 6 months.**

Effective contraception will be defined as regular and consistent use of oral contraceptive pills/patches/vaginal ring (Nuvaring®)/ injectable contraception/ intrauterine contraception or the contraceptive implant. Use of barrier contraception or other methods such as withdrawal methods or natural family planning will not be considered as effective contraception due to their higher relative failure rate.

At 6 months post-abortion, the Research nurse/Fellow will contact patients in both intervention and control arms to assess contraceptive uptake and continuation. Data will be collected using a standardized questionnaire (Appendix 2: Topic Guide E) to explore factors that could have potentially influenced contraceptive decision-making, compliance and continuation. Information will also be obtained about the number of contacts patients in both groups have had with Health Care professionals relating to contraception since their abortion.

- **Repeat abortion at 1 and 2 years**

Anonymised data using unique patient identifiers such as Date of Birth and post code will be used to identify the proportion of study participants in each arm undergoing a repeat abortion within 1 year and 2 years following their abortion. This data will be obtained from the data routinely collected by the Public Health Department in Lambeth from the three abortion provider units for Lambeth, Southwark and Lewisham. It is possible that a small proportion of the study participants may have moved outside the boroughs of Lambeth, Southwark and Lewisham over a period of 2 years following their abortion and may have presented elsewhere for their repeat abortion. The Department of Health routinely collects statutory data returns from all abortion providers in England and Wales on all legal abortions. We will attempt to obtain permission from the Department of Health for Data linkage with the national abortion dataset for more complete information on repeat abortion rates in our study participants.

**5. Ethics**

**5.1 Consent:**

Patients who have undergone an abortion may not wish to be contacted following the procedure for very personal reasons. Since the main focus of the study is to provide follow up to these women to facilitate contraceptive uptake and continuation, we plan to engage these women by obtaining a fully informed consent at the start of the study.

**5.2 Confidentiality:**

Patients will be given unique identifiers on entry to the study. To enable us to follow them up at 1 and 2 years to examine the repeat abortion rate without having to re-contact the patient, routinely collected data from the Public Health Department in Lambeth and the Department of Health will be matched to our research data using initials, date of birth and post codes. The link between the unique patient identifier and patient details will be maintained within a secure password protected NHS database, using all current guidelines employed by King’s College NHS Foundation Trust. Only NHS encrypted memory sticks will be used for data transfer. Once the database is locked at the end of the study period (3 years) all links will be destroyed by removing initials, dates of birth and postcodes from the study data.

**5.3 Vulnerable clients :**

**1) Victims of sexual assault:**

Victims of sexual assault undergoing an abortion will be included in the study as these women may benefit from specialist contraceptive support following their abortion and research information about the effect of any intervention to prevent future unplanned pregnancies in this group will be useful. In the event of disclosure of sexual assault, patients will also be offered a referral to dedicated Specialist services for victims of sexual assault for additional support.

**2) Under 16 year olds:**

We decided to include under-16’s in this study as they are a vulnerable group with a high abortion rate and research information about the effect of any intervention to prevent future unplanned pregnancies in this group is crucial. All under-16’s participating in the study will be assessed for Fraser competence before being given advice and treatment. They will also be assessed to explore any child protection issues that may arise and will be referred to the Safeguarding Children team as may be required. All clinicians involved in the research team are trained in Child protection procedures.

**3) Those who do not speak English:**

We have excluded non-English speaking patients from our study due to the logistics of obtaining informed consent and providing telephone follow-ups.

**4) Patients who lack capacity to consent for themselves:**

These patients will be excluded from the study.

### 6 Statistics

### 6.1 Sample Size

| A stratified design, which divides the sample among 6 strata (3 centres, 2 arms in each centre), is analyzed using the two-sided, Cochran-Mantel-Haenszel test. Sample sizes, summed across all strata, of 223 in intervention group and 223 in control group achieve 95% power and 0.05 significance to detect a difference of 15% in the percentage of women using LARC (Long- acting methods of reversible contraception such as injectables, implant or intrauterine device) or contraceptive pills in each group at 6 month follow-up. Allowing for 65% accepted participation rate and 60% follow-up rate, we require a total of 1144 patients across the three centres: 618 patients from BPAS, 366 from MSI and 160 patients from King’s College Hospital.    **Randomisation**: centre-stratified block randomization  **Primary outcome:** contraceptive uptake at 6months  **Secondary outcome:** repeat abortion rate at 1 year and 2 years  **Sample size calculation:**  Based on abortion data for Lambeth, Southwark and Lewisham residents in 2009, there were 2885 NHS abortions in BPAS, 1725 in MSI and 748 in King’s College Hospital. From this data, we can deduce the average of number of abortions at 3 months as: 721 (54%) abortions in BPAS, in 431 (32%) MSI and 187 (14%) in Kings with a total number of abortions for all the 3 centres estimated as 1339. It is anticipated that recruitment could be completed in 4-6 months.  Based on published papers (Schunmann et. Al [21]), there was 68% uptake of LARC or contraceptive pills at 4months following abortion; we have assumed the same at 6months post-abortion. 34.84% refused to participate in the above study; We have used 35 % refusal to participate for sample size calculation. There was 61.5% follow-up rate at 4months in the above study. We have used 60 % follow-up rates at 6 months. |
| --- |

**6.2 Randomisation**

| Please refer to section 3.4 |
| --- |

**6.3 Analysis**

**Statistical analysis of primary outcome:** comparison of the binary outcomes (rates) in the two arms and adjusting for centres using Cochran-Mantel-Haenszel test.

**Statistical analysis of Secondary outcome**: comparison of the proportions (rates) in the two arms, Chi2 test, logistic regression and time-to-event analysis.

|  |
| --- |

### 7. Data Monitoring Committee

| The data monitoring committee will comprise of the Chief Investigator, co-investigators the research nurse/fellow and the statistician and will meet 2 weekly during the initial months of the recruitment phase and subsequently 4 weekly to assess progress with recruitment, data collection, data entry, loss to follow-up rates and interim analyses. |
| --- |

### 8. Direct Access to Source Data and Documents

| The Investigator(s) will permit trial-related monitoring, audits, REC review, and regulatory inspections (where appropriate) by providing direct access to source data and other relevant documents. |
| --- |

### 9. Ethics & Regulatory Approvals

| The trial will be conducted in compliance with the principles of the Declaration of Helsinki (1996), the principles of GCP and in accordance with all applicable regulatory requirements including but not limited to the Research Governance Framework and the Medicines for Human Use (Clinical Trial) Regulations 2004, as amended in 2006 and any subsequent amendments.  This protocol and related documents will be submitted for review to King’s College Hospital Research Ethics Committee (REC) and Ethics committees of British Pregnancy Advisory Service and Marie Stopes International.  Annual progress and safety reports and a final report at conclusion of the trial will be submitted to the REC. |
| --- |

### 10. Quality Assurance

| Monitoring of this study to ensure compliance with Good Clinical Practice and scientific integrity will be managed and oversight retained by the Sexual Health and HIV Research Governance team at King’s College Hospital. |
| --- |

### 11. Data Handling

| The Chief Investigator will act as custodian for the research data.  Patients will be given unique identifiers on entry to the study. To enable us to follow them up at 1 and 2 years to examine the repeat abortion rate without having to re-contact the patient, routinely collected data from the Public Health Department in Lambeth and the Department of Health will be matched to our research data using initials, date of birth and post codes. The link between the unique patient identifier and patient details will be maintained within a secure password protected NHS database, using all current guidelines employed by King’s College NHS Foundation Trust. Only NHS encrypted memory sticks will be used for data transfer. Once the database is locked at the end of the study period (3 years) all links will be destroyed by removing initials, dates of birth and postcodes from the study data. |
| --- |

### 12. Publication Policy

| It is intended that the results of the study will be disseminated through publication in peer-reviewed scientific journals and presented at national and international conferences. |
| --- |

### 13. Financial Aspects

| Funding to conduct the research study is provided by the London Sexual Health Programme. |
| --- |

### 14. Signatures

______________________________________ 11/02/2011

Chief Investigator Date

Dr Usha Kumar

**15. Appendices**

**15.1 Appendix 1: Patient Information Sheet (Full information sheet and summary sheet) and Consent Form**

Department of Sexual Health & HIV

Camberwell Sexual Health Centre

100 Denmark Hill

London SE5 9RS

**Patient Information Sheet**

**Study Title: Randomised controlled study of the impact of provision of follow-up contraceptive support to women who have had an abortion.**

You are being invited to take part in a research study. Please take time to read the following information carefully and ask us if there is anything that is not clear or if you would like more information.

**What is the purpose of the study?**

The aim of this research is to see if specialist support after abortion helps women with their use of contraception to prevent unplanned pregnancies in future. Your participation would be invaluable in helping us find out whether Specialist support works or not. If it does work, this may benefit other women in your situation in future.

You will be eligible to take part in the study if :

**1. You are a resident of Lambeth/Southwark/Lewisham or have a GP in one of these boroughs.**

**and**

**2. You plan to live in United Kingdom for the next 6 months**

**What will happen if I take part?**

If you do decide to take part you will be asked to sign a consent form. You are still free to withdraw from the study at any time and without giving a reason. A decision to withdraw at any time, or a decision not to take part, will not affect the standard of care you receive.

We will collect some personal information eg. date of birth, ethnicity, postcode, education, relationship status, number of previous pregnancies, contraception used.

**Study treatment**

You will be allocated by chance to either the standard care group or the specialist support group. Everyone has an equal chance of being in either group; you will not be allowed to choose and will not be told which group you have been assigned to at the time of enrolment into the study. This ensures that both groups are similar, so that if there are any differences between how the groups do, we know it will be due to the specialist support.

**Standard care group**: If you are placed in this group, your follow up care after abortion will remain the same as is current standard practice. You will be given full information about contraceptive choices available and where to access them.

**Specialist support Group:** If you are placed in this group, in addition to the standard contraceptive care you receive, you will be offered a consultation with a contraception specialist doctor/nurse from King’s College Hospital at:

- 2-4 weeks after your abortion (telephone/face-face clinic appointment) lasting about 30 minutes. During this consultation, the specialist will try to help you choose a contraceptive method that suits you or help you with any problems you may have with a contraceptive method you are already using. If you miss your appointment we will ring you to offer another suitable appointment.
- 3 months after your abortion: Telephone interview with the research doctor/nurse lasting about 15 minutes to address any problems you may have with your chosen method of contraception and offer help with changing the method if you wish.

**Both groups** will receive a telephone follow up lasting about 15 minutes with the research doctor/nurse 6 months after abortion to see how you are doing with your contraception.

**You will not be paid to take part in the study.**

**Study follow-up**

We will be studying routinely collected data on abortions to see if there are any repeat abortions in the study participants during the 2 year period following the abortion. To do this, we will be linking individual study data with information on abortions routinely collected by the Department of Health. This will not involve you as we do not need to contact you after the telephone call at 6 months.

**What if there is a problem?**

If you have any concerns or problems in relation to your participation in the study you may contact the Chief Investigator, Dr.Usha Kumar on 0203 299 5043 or 0203 299 5041 at King’s College Hospital NHS Foundation Trust and your concerns will be addressed individually.

**Will my taking part in this study be kept confidential?**

All information which is collected about you during the course of the research will be kept strictly confidential. However, there will be limits to confidentiality should something of concern be disclosed.

If you agree to join the study, the data collected for the study will be looked at by authorised persons from the Research team.

**What will happen to the results of the research study?**

The results of the study will be published in a medical journal and may be presented at medical conferences. You will not be identified in any study report or publication.

A summary of the overall study results for patients who have participated will be produced once the study has been completed and will be available to you .

**Contact for Further Information**

If you have any further questions about this study please discuss them with your doctor/nurse. If you would like further information on this study please ask:

Dr. Usha Kumar (Chief Investigator) who can be contacted at 0203 299 5043 or 0203 299 5041 or e-mail: [u.kumar@nhs.net](mailto:u.kumar@nhs.net)

**The research is funded by the London Sexual Health Programme, and is sponsored and regulated by King’s College Hospital NHS Foundation Trust.**

**The study has been approved by the Research Ethics Committee.**

**Once again, we would like to thank you for taking the time to read this information and for considering taking part in this study.**

Department of Sexual Health & HIV

Camberwell Sexual Health Centre

King’s College Hospital Foundation Trust

100 Denmark Hill

London SE5 9RS

**Study title**

Randomised controlled study of the impact of provision of follow-up contraceptive support to women who have had an abortion.

**Summary Patient Information Sheet**

You are being invited to take part in the above research study to look at the effectiveness of providing follow up support to women after they have undergone an abortion to help them with their contraception. If you agree to take part in the study, you will be allocated by chance to one of 2 groups:

**Standard care group**: If you are allocated to this group, your follow up care after abortion will remain the same as is current standard practice. You will be given full information about contraceptive choices available and where to access them from.

**Specialist support group:** If you are allocated to this group, in addition to the standard contraceptive care you receive, you will be offered a consultation by telephone or clinic appointment at King’s College Hospital with a contraception specialist doctor/nurse between 2-4 weeks after your abortion and a further telephone follow-up after 3 months to help you with your contraception.

**Both groups** will receive a telephone follow up with the research doctor/nurse 6 months after abortion to see how you are doing with your contraception.

We will also be studying routinely collected data on abortions to see if there are any repeat abortions in the study participants during the 2 year period following the abortion. This will not involve you as we do not need to contact you after the telephone call at 6 months.

This study will help us find out if the specialist support helped women to effectively use contraception and prevent further unplanned pregnancy. If such follow up specialist support proves to be beneficial, we will make recommendations for this to be made standard practice.

**PLEASE READ THE FULL PATIENT INFORMATION SHEET FOR FURTHER DETAILS**


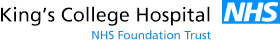


Department of Sexual Health & HIV

Camberwell Sexual Health Centre

100 Denmark Hill

London

SE5 9RS

**Centre Identifier:**

**Patient Identification Number for this trial:**

**Study Title:** Randomised controlled study of the impact of provision of follow-up contraceptive support to women who have had an abortion

**Chief Investigator:** Dr Usha Kumar

**CONSENT FORM**

**Please initial box to agree**

| 1.     I confirm that I have read and understand the information sheet dated 26th April 2011 (Version 7.0) for the above study and have had the opportunity to ask questions and discuss it with my health care professional. |  |
| --- | --- |
| 2.     I understand that my participation is voluntary and that I am free to withdraw at any time, without giving any reason, without my medical care or legal rights being affected. |  |
| 3.     I understand that sections of my medical notes may be looked at by responsible individuals involved in the running of the study or from regulatory authorities where it is relevant to my taking part in research. I give permission for these individuals to have access to my records. |  |
| 1. I understand that my participation in the study will involve access to information routinely held by the Department of Health. 2. I agree to be contacted on these telephone numbers:   Mobile: _________________________ Landline: _____________________   1. I agree to take part in the above study. 2. **(Optional)** I agree to my GP/relevant healthcare professional being informed of my participation in this study. 3. **(Optional)** I agree to be contacted in future for participation in research on a related topic. |  |
|  |  |

Name of participant: __________________________________________________

Date: ___________________ Signature: _________________________

Name of person taking consent: ________________________________________

Date: ____________________ Signature: _________________________

Name of researcher: ___________________________________________________

Date: ____________________Signature: _________________________

**3 copies:** 1 for patient, 1 for researcher, 1 to be kept with hospital notes

**15.2 Appendix 2: Topic Guides for data collection**

1. **Topic Guide for baseline data collection at recruitment**: (This will be collected for both the ‘Control’ and ‘Intervention’ groups)

- Date of Birth
- Ethnicity
- Post code
- Educational achievement
- Marital/Relationship status
- Parity
- Previous abortions
- Contraceptive use immediately prior to current pregnancy
- Planned method of contraception after abortion
- Who referred for the abortion : Self/GP/Family Planning or Sexual Health clinic/other

1. **Topic Guide for Data collection at the 2-4 week consultation with the Specialist in Reproductive and Sexual Health:** (This is for the ‘Intervention’ Group only)

- Any change in the relationship status
- Whether accompanied by partner for this visit (if having a face-face consultation in a clinic)
- Smoking
- Alcohol use
- Drug misuse
- Domestic violence
- Relevant Medical History
- Any physical problems following the abortion
- Level of psychological distress following abortion
- Any additional psychological support required
- Any concerns about future fertility
- Any unprotected sexual intercourse since the last abortion
- Any use of Emergency contraception since the last abortion
- Contraceptive method provided at the time of abortion
- Current method of contraception
- When was the method started
- Is the contraceptive method being effectively used
- Any side-effects from the contraceptive method
- Any particular method of contraception medically contraindicated
- Previous contraceptive use
- Any problems with previous contraceptive methods
- Any particular contraceptive method/s unacceptable to the patient
- Attitude to future unplanned pregnancy
- Awareness of safer sex practice, STIs
- Contraceptive method provided at this visit
- Arrangements for Follow up
- Any referral to other agencies/other Healthcare professionals:
- Social services
- Safeguarding children team
- Safeguarding adults team
- Domestic violence support
- Alcohol and Drug misuse
- Mental Health team
- Psychologist
- Health advisor/counsellor
- Haven
- Services for trafficked women
- Support services for women working in prostitution
- General Practitioner

1. **Topic Guide for the phone call to study participants who DNA the 2-4 week appointment** (This is for the Intervention group only)

- Reason for DNA
- Offer suitable alternative appointment (telephone consultation or face-face clinic appointment) as preferred by the patient
- Telephone assessment (as in Section B) if willing
- Telephone Intervention purely focussed on facilitating contraceptive uptake if not willing for all the details in Section B
- Link with other Health Care professional as necessary

1. **Topic Guide for the phone call to study participants at 3 months post abortion** (This is for the intervention group only)

- Change in the relationship status
- Contact with GP or contraception service for contraceptive advice between the 2 week appointment and now
- Current method of contraception
- When was the method started
- Is the contraceptive method being effectively used
- Any side-effects from the contraceptive method
- Any unprotected sexual intercourse since the recent abortion
- Any use of Emergency contraception since the recent abortion
- Any further support required
- Arrangements for follow up

1. **Topic Guide for the phone call to study participants at 6 months post abortion** (This is for both the ‘Intervention’ and ‘Control’ group)

- Any change in the Relationship status
- Current method of contraception
- When was the method started
- Is the contraceptive method being effectively used
- Patient satisfaction with the contraceptive method
- Any unprotected sexual intercourse since the recent abortion
- Any use of Emergency contraception since the last abortion
- Any contact with GP/contraception service for contraceptive advice between the abortion and now
- Pregnancy intentions for the next 1 and 2 years
- Attitude to future unplanned pregnancy
- Alcohol use
- Drug misuse
- Domestic (Intimate partner) violence

______________________________________________________________________________________
